# The systemic inflammatory response, performance status and survival in patients undergoing alpha-interferon treatment for advanced renal cancer

**DOI:** 10.1038/sj.bjc.6602152

**Published:** 2004-08-31

**Authors:** E Bromwich, D C McMillan, G W A Lamb, P A Vasey, M Aitchison

**Affiliations:** 1 University Department of Surgery, Royal Infirmary, Glasgow, G31 2ER, UK; 2 Department of Urology, Gartnavel General Hospital, Glasgow, G12 0YN, UK; 3 Department of Medical Oncology, Beatson Oncology Centre, Glasgow, G11 6NT. UK

**Keywords:** systemic inflammatory response, C-reactive protein, performance status, survival, *α*-interferon, renal cancer

## Abstract

The prognostic value of C-reactive protein, compared with ECOG performance status (ECOG-ps), in patients receiving alpha-interferon treatment for advanced renal cancer was assessed in 58 patients. In all, 55 patients died on follow-up. On multivariate analysis with ECOG-ps and C-reactive protein entered as covariates, only C-reactive protein was a significant independent predictor of survival (HR 2.03, 95% CI 1.09–3.80, *P*=0.026).

Renal cell cancer continues to be one of the most lethal urological tumours. Although only 2% of the population develop renal cancer, one-third of these patients will have metastatic disease at presentation and a further 40% will develop metastases during the course of their disease, with an expected survival in the order of 8–10 months (Montie, 1994; World Health Organisation, 2000).

For the majority of these patients, immunotherapy remains the treatment of choice; in the UK, alpha-interferon has become the mainstay of treatment. The largest randomised trial comparing alpha interferon with the existing hormonal drug medroxyprogesterone acetate demonstrated a statistically significant survival advantage for the interferon group (MRC RCC, 1999).

There is increasing evidence that the presence of a systemic inflammatory response, as evidenced by an elevated circulating C-reactive protein concentration, is an independent prognostic factor in a number of hormone-independent cancers (Mahmoud and Rivera, 2002; Forrest *et al*, 2003; McMillan *et al*, 2003). Two studies have shown that C-reactive protein has prognostic value in patients with renal cell cancer, but was not independent of disease stage (Ljungberg *et al*, 1995; Miyata *et al*, 2001). These studies included patients with stage I disease, who were unlikely to progress, and patients with stage IV disease, who had already progressed, and this may have confounded the assessment of the prognostic value of an elevated circulating C-reactive protein concentration. Indeed, Blay *et al* (1992) reported that, in patients with metastatic renal cancer receiving interleukin-2 immunotherapy, a high C-reactive protein concentration was associated with poor survival independent of performance status.

The aim of the present study was to examine the relationship between the systemic inflammatory response, performance status and survival in patients undergoing alpha interferon immunotherapy for advanced renal cancer.

## PATIENTS AND METHODS

### Patients

Patients with advanced renal cell cancer at a single multi-disciplinary clinic in the North Glasgow NHS Hospital Trust between August 1997 and October 2002 were studied. Information was abstracted from the case notes (EB). Data for 1997–1999 were collected retrospectively (*n*=26) and that for 2000–2002 prospectively (*n*=32).

Prior to immunotherapy, patients were assessed by a CT scan of the chest, abdomen and pelvis, and where appropriate a bone scan; performance status (ECOG-ps) was also assessed and a blood sample was taken for measurement of C-reactive protein concentration.

Patients were treated with subcutaneous alpha-interferon at a dosage of 10 MIU three times weekly. Patients were followed up weekly. After 12 weeks of treatment, a further CT scan was performed and response was assessed. Patients with stable or responding disease continued on alpha-interferon if their tolerance was acceptable.

Routine laboratory measurements of C-reactive protein concentration were carried out. C-reactive protein was measured by fluorescence polarisation immunoassay using a TDX analyser and reagents obtained from Abbott Laboratories (Abbott Park, IL, USA). The limit of detection of the assay is a C-reactive protein concentration of <5 mgl^−1^. The coefficient of variation for this method, over the range of measurement, was less than 5%, as established by routine quality control procedures.

### Statistics

C-reactive protein concentrations were grouped either as ⩽10/>10 mgl^−1^ (O'Gorman *et al*, 2000) or as ⩽50/>50 mgl^−1^ (Blay *et al*, 1992) as described previously. Univariate survival analysis was performed using the Kaplan–Meier method with the logrank test. Multivariate survival analysis and calculation of hazard ratios (HR) were performed using Cox regression analysis. Deaths up to 31 January 2004 were included in the analysis. Analysis was performed using SPSS software (SPSS Inc., Chicago, IL, USA).

## RESULTS

The characteristics of patients with advanced renal cancer receiving alpha-interferon immunotherapy (*n*=58) are shown in Table 1

**Table 1:** Clinical characteristics and survival in patients with advanced renal cancer receiving alpha interferon immunotherapy: univariate survival analysis

	**Patients 58 (100%)**	**Survival (months) Median (95% CI)**	***P*-value**
*Age* (*years*)			
⩽60	28 (48)	10.6 (6.3–15.0)	
>60	30 (52)	12.6 (5.9–19.4)	0.774
			
*Sex*			
Male	41 (71)	13.5 (3.9–23.0)	
Female	17 (29)	12.2 (9.0–15.4)	0.831
			
*Performance status (ECOG)*			
0	11 (19)	16.2 (7.2–25.2)	
1	40 (69)	13.5 (10.8–16.1)	0.046
2	7 (12)	4.3 (2.3–6.2)	
			
*C-reactive protein (mgl*^−*1*^)			
⩽10	17 (29)	15.9 (2.4–29.5)	
>10	41 (71)	10.1 (4.2–16.1)	0.023
			
*C-reactive protein (mgl*^−*1*^)			
⩽50	33 (57)	14.1 (10.0–18.2)	
>50	25 (43)	5.4 (2.4–8.5)	0.073
			
*Treatment (weeks)*			
⩽12	27 (46)	5.1 (4.4–5.8)	
>12	31 (54)	18.6 (14.5–22.7)	<0.001

. The majority were male and over the age of 60 years. Approximately 90% had an ECOG performance status of 0–1, 70 and 50% had a C-reactive protein >10 and >50 mgl^−1^, respectively. Approximately half of the patients had a treatment duration more than 12 weeks.

The minimum follow-up was 36 months and 55 (95%) patients died during the follow-up period. On univariate survival analysis, ECOG-ps (*P*<0.05), C-reactive protein (<10/>10 mgl^−1^, *P*<0.05) and treatment duration (*P*<0.001) were significant predictors of survival. On multivariate analysis with ECOG-ps and C-reactive protein entered as covariates, only C-reactive protein was a significant independent predictor of survival (HR 2.03, 95% CI 1.09–3.80, *P*=0.026). The cancer-specific survival rates at 3 years for patients with a C-reactive protein ⩽10/>10 mgl^−1^ were 24 and 5%, respectively.

Of those patients with an elevated C-reactive protein concentration (>10 mgl^−1^) 44% received alpha-interferon immunotherapy for more than 12 weeks compared with 76% of those with a C-reactive protein concentration in the normal range (*P*=0.041, Fisher's exact test).

## DISCUSSION

Conventionally, in patients with advanced renal cancer, the decision whether or not to offer immunotherapy is primarily based on performance status. However, the assessment of performance status reflects functional status at a specific point in time and remains subjective. For example, significant differences in the assessment of performance status have been reported between oncologists, nurses and patients, oncologists being the most optimistic in their assessment and patients the least (Ando *et al*, 2001). There is therefore a continuing interest in prognostic factors, which better reflect response to treatment and survival (Bennett and Ryall, 2000; Sloan *et al*, 2001).

**Figure 1:**
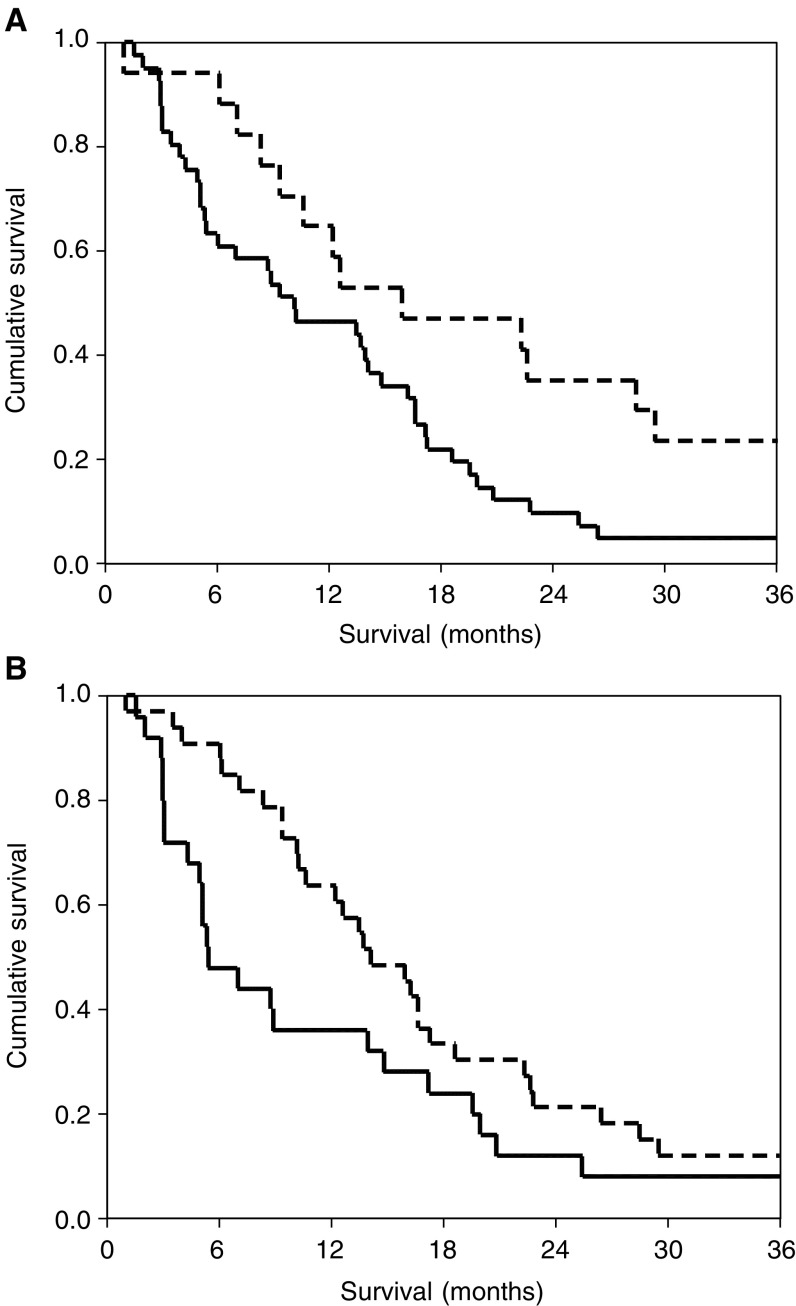
(**A**) The relationship between C-reactive protein (⩽10 mgl^−1^ - - -, >10 mgl^−1^ **__**) and survival in patients receiving alpha-interferon treatment for advanced renal cancer. (**B**) The relationship between C-reactive protein (⩽50 mgl^−1^ - - -, >50 mgl^−1^ **__**) and survival in patients receiving alpha-interferon treatment for advanced renal cancer.

In the present study, we compared the prognostic value of performance status and the systemic inflammatory response (using two C-reactive protein cutoffs, ⩽10/>10 mgl^−1^ and ⩽50/>50 mgl^−1^) in patients receiving alpha-interferon immunotherapy for advanced renal cancer (Figure 1

). In this small study, on univariate analysis, both ECOG-ps and C-reactive protein (<10/>10 mgl^−1^ only) had significant prognostic value. However, when compared in multivariate analysis, only C-reactive protein retained independent significance and therefore it would appear that it is superior to performance status in predicting survival following alpha-interferon immunotherapy.

These results are consistent with previous studies, which have shown that the presence of a systemic inflammatory response (C-reactive protein >10 mgl^−1^) is associated with poor outcome independent of clinical stage and performance status (O'Gorman *et al*, 2000; Scott *et al*, 2002; Forrest *et al*, 2003) and is superior to performance status in predicting survival following chemotherapy (Forrest *et al*, 2004).

The side effects of alpha-interferon immunotherapy are significant and include a flu-like syndrome, fatigue, fever, anorexia, myalgia, arthralgia, dry skin and mucous membranes, mental state changes and depression. In the present study, although approximately 90% of patients had an ECOG-ps of 0 or 1, 31 (53%) patients completed 12 weeks of therapy, with five (9%) patients showing a complete or partial response, consistent with the response rate (14%) in the MRC trial (MRC RCC, 1999). It was therefore of interest that, in the present study, of those patients with an elevated C-reactive protein concentration only 44% received alpha-interferon immunotherapy for more than 12 weeks compared with 76% of those with a C-reactive protein concentration in the normal range. This may explain, in part, the effect of the systemic inflammatory response on the poor survival of patients with advanced renal cancer receiving alpha-interferon immunotherapy.

The underlying basis of the relationship between the presence of a systemic inflammatory response and poor tolerance to alpha-interferon immunotherapy or chemotherapy (Forrest *et al*, 2004) is not clear. However, it is likely to be linked to increased toxicity in these patients. Therefore, it is of interest that the activity of the enzyme cytochrome *P*450 3A, involved in biotransformation of more than half of all drugs currently available, is compromised in patients with an elevated C-reactive protein concentration (Rivory *et al*, 2002; Slaviero *et al*, 2003). It may be that moderation of the systemic inflammatory response will reduce such toxicity.

In summary, the prognosis from advanced renal cancer is very poor with or without immunotherapy. The presence of a systemic inflammatory response (C-reactive protein >10 mgl^−1^) appears to be a useful clinical indicator of poor outcome independent of performance status. This may assist in more appropriate selection of patients for treatment with alpha-interferon, thus reducing the potentially deleterious impact on an already shortened lifespan.
